# lncRNA HOTAIR overexpression induced downregulation of c-Met signaling promotes hybrid epithelial/mesenchymal phenotype in hepatocellular carcinoma cells

**DOI:** 10.1186/s12964-020-00602-0

**Published:** 2020-07-11

**Authors:** Hande Topel, Ezgi Bagirsakci, Dehan Comez, Gulsun Bagci, Gulcin Cakan-Akdogan, Nese Atabey

**Affiliations:** 1 Izmir Biomedicine and Genome Center (IBG), Balcova, 35340 Izmir, Turkey; 2 Department of Medical Biology and Genetics, Graduate School of Health Sciences, Dokuz Eylul University, 35340, Balcova, Izmir, Turkey; 3 Functional Genomics and Metabolism Unit, Department for Biochemistry and Molecular Biology, University of Southern Denmark, Campusvej 55, 5230 Odense M, Denmark; 4 Department of Molecular Biology and Genetics, Izmir International Biomedicine and Genome Institute, Dokuz Eylul University, 35340, Balcova, Izmir, Turkey; 5 Department of Medical Biology, Faculty of Medicine, Dokuz Eylul University, 35340, Balcova, Izmir, Turkey

**Keywords:** HCC, long non-coding RNA, HOTAIR, c-Met, Caveolin-1, Hybrid E/M

## Abstract

**Background:**

Epithelial-to-mesenchymal transition (EMT) and mesenchymal-to-epithelial transition (MET) are both reversible processes, and regulation of phenotypical transition is very important for progression of several cancers including hepatocellular carcinoma (HCC). Recently, it is defined that cancer cells can attain a hybrid epithelial/mesenchymal (hybrid E/M) phenotype. Cells with hybrid E/M phenotype comprise mixed epithelial and mesenchymal properties, they can be more resistant to therapeutics and also more capable of initiating metastatic lesions. However, the mechanisms regulating hybrid E/M in HCC are not well described yet. In this study, we investigated the role of the potential crosstalk between lncRNA HOTAIR and c-Met receptor tyrosine kinase, which are two essential regulators of EMT and MET, in acquiring of hybrid E/M phenotype in HCC.

**Methods:**

Expression of c-Met and lncRNA HOTAIR were defined in HCC cell lines and patient tissues through HCC progression. lncRNA HOTAIR was overexpressed in SNU-449 cells and its effects on c-Met signaling were analyzed. c-Met was overexpressed in SNU-398 cells and its effect on HOTAIR expression was analyzed. Biological significance of HOTAIR/c-Met interplay was defined in means of adhesion, proliferation, motility behavior, invasion, spheroid formation and metastatic ability. Effect of ectopic lncRNA HOTAIR expression on phenotype was defined with investigation of molecular epithelial and mesenchymal traits.

**Results:**

In vitro and in vivo experiments verified the pivotal role of lncRNA HOTAIR in acquisition of hybrid E/M phenotype through modulating expression and activation of c-Met and its membrane co-localizing partner Caveolin-1, and membrane organization to cope with the rate limiting steps of metastasis such as survival in adhesion independent microenvironment, escaping from anoikis and resisting to fluidic shear stress (FSS) in HCC.

**Conclusions:**

Our work provides the first evidence suggesting a role for lncRNA HOTAIR in the modulation of c-Met to promote hybrid E/M phenotype. The balance between lncRNA HOTAIR and c-Met might be critical for cell fate decision and metastatic potential of HCC cells.

**Video Abstract**

## Background

Liver cancer is ranked as 7^th^ cancer type in the list of cancers with the highest number of cases incident between 2007 and 2017 by Global Burden of Disease (GBD) 2017 study. Hepatocellular Carcinoma (HCC) accounts more than 80% of liver cancer cases and it is estimated to be the 4^th^ most common cause of cancer related deaths worldwide [1]. HCC is reported to have complex and heterogeneous molecular features which is previously stressed out by integrative studies combining genomic characterization, exome sequencing, transcriptome analysis and clinical trials. Molecular heterogeneity and aetiological complexity of HCC makes it unlikely for one treatment or agent to effectively target all or most HCCs [2, 3].

c-Met, a receptor tyrosine kinase, is known to be upregulated in liver diseases favoring hepatocyte proliferation. Besides potential benefits in chronic liver diseases, c-Met contributes to initiation, development and progression of HCC. c-Met activation in HCC is mostly driven by molecular networks instead of activating mutations and it is activated by non-canonical signaling mechanisms as well as canonical activation by its ligand, hepatocyte growth factor (HGF). c-Met is regarded as one of the most promising targets for HCC treatment and c-Met targeted clinical trials are being conducted, currently [4]. In addition to its contribution to HCC development and progression, c-Met is also considered to be a key player in drug resistance [5].

HOX transcript antisense intergenic RNA, HOTAIR, is a 2148-nt-long spliced and polyadenylated long non-coding RNA (lncRNA) encoded within HOXC cluster. HOTAIR is first defined to take role in epidermal tissue development by recruiting chromatin remodeling complexes to its epigenetic targets [6, 7]. Overexpression of HOTAIR has been associated with poor prognosis, invasiveness and aggressiveness of various cancer types [8–11]. Complex secondary structure and ability to form independent structural domains ensure the multi-acting nature of HOTAIR and it has been defined to contribute to various cellular mechanisms via different molecular interactions such as scaffolding protein complexes, decoying microRNAs, epigenetically targeting genes and enabling RNA-protein/DNA-protein interactions [7, 12–14].

Transcription factors, non-coding RNAs and epigenetic regulators play critical roles in cell-fate determination such as transition between epithelial and mesenchymal phenotypes. Starting from the origin of a tumor, cancer cells go through complex and dynamic phenotypical changes -epithelial to mesenchymal transition (EMT), or its reverse mesenchymal to epithelial transition (MET)- to cope with metastasis rate-limiting steps. It has been reported that, some cancer cells display both epithelial and mesenchymal markers and this phenotype is referred as hybrid, intermediate, partial, metastable or incomplete EMT phenotype [15–18]. The tumor cells simultaneously expressing both mesenchymal and epithelial markers may be more plastic and most likely to contribute to metastatic outgrowth. Cells bearing this hybrid phenotype are first defined in circulating tumor cells (CTCs) of patients with both epithelial and mesenchymal traits [19, 20]. Hybrid cells that co-express mesenchymal and epithelial markers migrate collectively, more resistant to fluidic shear stress damage in circulation, exit from anoikis and have enhanced metastatic ability than cells with complete epithelial or mesenchymal phenotypes [21]. That is why, cancer cells that attain a hybrid E/M (epithelial/mesenchymal) phenotype pose greater risk for metastasis in cancer patients [22].

c-Met is reported to play an essential and complex role in modulation of transitional states within the broad spectrum of cellular phenotypes [23]. Although HOTAIR is also reported to contribute attainment of cellular phenotypes, its role is generally defined in means of correlations with aggressiveness traits of cancer cells [10, 11, 24, 25]. As the number of the studies reporting induced expression and critical roles of these two molecules in HCC are increasing, the likelihood of their direct or indirect molecular interaction becomes non-negligible. In this study, we aimed to investigate the nature of their interaction and here, we describe the interplay between HOTAIR and c-Met in HCC context. Our data present strong evidence that HOTAIR ensures hybrid E/M phenotype and its downregulation is required for c-Met induced complete mesenchymal phenotype in HCC cells.

## Materials and methods

### Cell culture

All Human HCC cell lines were kindly provided by Prof. Dr. Mehmet Öztürk (Izmir Biomedicine and Genome Center, Izmir, Turkey). FOCUS, SNU-449, SK-HEP-1, SNU-475, SNU-387, SNU-423, SNU-398, MAHLAVU, HEP-3B and HuH-7 cell lines were cultured as described previously [26]. All cell lines were tested for mycoplasma infection and confirmed as negative before the experiments. SU11274 (Calbiochem, 448101) is solubilized with DMSO and used with 2500 nM concentration. c-Met kinase activity was inhibited with SU11274 (2500 nM in 2% FBS complete medium). Ligand dependent c-Met activation was induced by 10 ng/ml Hepatocyte Growth Factor (HGF) in 2% FBS complete medium (R&D system, USA). Sorafenib treatment was continuously applied with the amount of their growth inhibition 50 (GI50) values (6 μM of Sorafenib) to MAHLAVU sorafenib resistant cell clones which have been established in our previous studies [5].

Cells were sheared with 205 U/CA multichannel peristaltic pump and Marprene tube elements of 2.79 bore for tubing (Watson Marlow, UK). Adherent cells were detached with 0.25% Trypsin /EDTA (Biological Industries, #03–050-1B, Israel) and 3 million cells in 8 ml of culture media were cultured in circulation under 0.5 dyn/ cm^2^ shear stress at 37 °C, in humidified incubator supplied with 5% CO_2,_ for 1 hour. After FSS application, cells were stained with trypan blue and counted with hemocytometer to evaluate cell viability under shear stress.

### mRNA isolation, reverse transcription and RT-qPCR

NucleoSpin RNA Isolation kit (Macherey-Nagel, Germany) has been used for mRNA isolation. mRNA isolation performed according to the manufacturer’s instructions. mRNA eluted with DNAse/RNAse free distilled water and its concentration is measured via NanoDrop.

SensiFast™ cDNA Synthesis Kit (Bioline Meridian Bioscience, USA) were used to transcribe cDNA from mRNA. qPCR reactions were performed with primers designed specific for cDNA sequences of gene of interests (CDS) from NCBI-Gene Bank. RT-qPCR analysis performed using SensiFast™ SYBR No-ROX kit (Bioline Meridian Bioscience, USA) with ABI 7500 Fast Real-Time PCR System (Thermo Scientific, USA) according to the manufacturer’s instructions. RPL41 and/or GAPDH gene expressions were used as internal controls to normalize relative expression of the genes. Primers targeting mRNAs of interest were listed in Supplementary Document 1.

### siRNA gene silencing experiments

HuH-7 cells were transfected with 10 nM non-targeting (untargeted, negative control-NC, Dharmacon, Lincode non-targeting SMARTpool, D-001320-10-50) and HOTAIR targeting siRNAs (Dharmacon, Lincode Human HOTAIR SMARTpool, R-187951-00-0050) by Roche X-tremeGENE™ HP DNA Transfection Reagent (6366244001, Roche). Cells were harvested after 48 h post-transfection and processed for further protein and mRNA investigations.

### Fluorescent In-Situ Hybridization (FISH)

HOTAIR mRNA was hybridized with Stellaris® FISH Probes, Human HOTAIR with Quasar® 570 Dye (LGS Biosearch Technologies, VSMF-2178-5). HCC cells were grown on glass coverslips and fixed with 3:1 methanol-acetic acid (MeOH-AcOH) fixation solution for 10 min. Hybridization was performed according to the manufacturer’s instructions with 500 nM HOTAIR targeting probes for 4 h at 37 °C. Cell nucleus was stained with DAPI.

Labeling with c-Met antibody and hybridizing HOTAIR mRNA with fluorescent-labeled probes were performed sequentially. For sequential labeling, HCC cells were firstly fixed with 3.7% (vol./vol.) formaldehyde in 1X-PBS for 10 min at room temperature. Fixed cells were incubated with primary antibody against c-Met protein (Cell Signaling, #3127) for 2 h at room temperature. After couple of washes with 1X-PBS, cells were incubated with secondary antibody (Alexa488-conjugated goat anti-mouse secondary antibody, Invitrogen, A-11001) for 1 h at RT. After immunofluorescent labeling, cells were fixed again with 3:1 methanol-acetic acid (MeOH-AcOH) fixation solution for 10 min. Following hybridization was performed according to the manufacturer’s instructions with 500 nM HOTAIR targeting probes for 4 h at 37 °C. Cell nucleus was stained with DAPI and cells were analyzed with Zeiss LSM 880-Confocal Laser Scanning Microscopy with Airyscan in IBG Optic Imaging Core Facility.

### Immunoblotting

The cells were lysed using RIPA lysis buffer containing 1 mM Na3VO2, 1 mM NaF, and 1% protease inhibitor cocktail (Roche Diagnostics, Indianapolis, IN, USA), and the lysates were subjected to Western blot analysis as described previously [26]. Primary antibodies are listed in Supplementary Document 1.

### Producing retroviral and lentiviral virus particles and generation of stable cell lines

SNU-449 cells were infected with retroviral vector bearing viruses. Retroviral vector bearing virus particles produced by transfection of HEK-293T cells with VSV-G envelope expressing plasmid (5 μg), gag-pol expressing plasmid (5 μg) and retroviral target vectors (5 μg) together by Roche X-tremeGENE™ HP DNA Transfection Reagent (6366244001, Roche). After 24 h of transfection, cell culture media refreshed and collected 48 h post-transfection. HCC cells were infected with 1:1 titration of retrovirus containing media. After 48 h, infected SNU-449 cells were selected with 13 μg/ml and SNU-398 cells were selected with 0.5 μg/ml puromycine (Life Technologies, #A1113803, USA) for at least 3 passages.

HEK-293T cells were transfected with vectors bearing Rev (1 μg), gag-pol (6 μg) and VSV-G envelope (3 μg) genes with lentiviral target vectors (2.5 μg) by transfection reagent. After 48 h, cell culture media collected and SNU-398 cells were infected with 1:5 titration of lentivirus containing media. After 48 h of infection, infected cells were selected with 0.5 μg/ml puromycine for at least 3 passages. Lentiviral and retroviral vectors and their origins are listed in Supplementary Document 1.

### Immunofluorescent analysis of the cells

HCC cells were grown on glass cover-slips and after experimental conditions set, cells were immunofluorescently labeled as described in previous study [27]. Imaging was performed with upright fluorescence microscope (Olympus - BX61) and Zeiss LSM 880-Confocal Laser Scanning Microscopy with Airyscan at IBG Optic Imaging Core Facility.

### Trans-well motility and invasion assays

Trans-well inserts with 8 μm pore size (SPL Life Sciences, #37224, Korea) were used to analyze motility and invasion ability of HCC cells. The mix of 0.25 mg/ml basal membrane extract (Corning Matrigel® Growth Factor Reduced (GFR) Basement Membrane Matrix, Phenol Red-Free, #356231) was coated on invasion inserts. Trans-well motility and invasion experiments performed as described in previous study [26]. Motile and invasive cells located at the exterior surface of the inserts were counted with bright field inverted microscope and statistical analysis was performed via Prism 8.

### Real-time adhesion, proliferation, motility and invasion assays

Real-time cell growth monitoring was performed with the Real-Time Cell Analyzer, xCELLigence System (Roche, Germany) as described previously [28]. 5000 cell/well was plated to analyze proliferation and adhesion to cell-culture treated wells of E-Plates. For motility and invasion assays, CIM-plates (ACEA Biosciences, # 0566581701, USA) were used with xCELLigence System. CIM-plate wells were coated with 0.25 mg/ml basal membrane extract (Corning Matrigel® Growth Factor Reduced (GFR) Basement Membrane Matrix, Phenol Red-Free, #356231) for invasion assay. For motility and invasion assays, 30.000 cells/well were plated to CIM-Plate wells and incubated for 24 h by monitoring with xCELLigence RTCA system.

### Hanging drop spheroid formation assay

2500 HCC cells were incubated within 20 μl culture medium drops which were hanging from the inverted lid of 10 cm cell culture plates. The reservoir part of 10 cm of tissue culture dish was filled with 3–5 ml of 1X PBS to provide a humidified environment. The culture dish was incubated at 37 °C, 5% CO2, 95% humidified incubator for 72 h or 96 h until the spheroids formation and spheroids were imaged under Olympus SZX10 stereo microscope and SC50 camera system.

### 2D Colony formation assay

SNU-449 HOTAIR OE and SNU-449 MOCK cells seeded on 24-well plates as 70 cells/well. The cells were cultured until single-cell colony formation. Cell culture media was refreshed in every three days and cells were incubated for one week. The day of analysis, cell culture media was aspirated and colonies were fixed with 1:1 cold Aceton:Methanol solution at -20 °C for 10 min. Colonies stained with 20% Giemsa solution for 20 min. After washing with distilled water, plate was incubated at room temperature for air dry. Plates were imaged under BIO-RAD Gel Doc™ XR+ Gel Documentation System and the images were analyzed via ImageJ - ColonyArea plug-in [29].

### Phalloidin staining

HCC cells were seeded on glass cover slips (22 × 22 cm) and incubated overnight for adhesion. Phalloidin (Abcam, abID#176756, USA)staining was performed as previously described [30]. Imaging performed at IBG Optic Imaging Core Facility with fluorescent (Olympus - BX61) and Zeiss LSM 880-Confocal Laser Scanning Microscopy with Airyscan.

### MTT assay

SNU-449 HOTAIR OE and SNU-449 MOCK 1000 cells per well were seeded on 96-well plate and incubated for 24, 48 and 72 h. MTT analysis was performed as described previously [31]. Measured absorbance was analyzed by Microsoft Excel 2016 and plotted by Prism 8.

### Analysis of TCGA and GEO microarray datasets

Normalized gene expression data from dataset GSE89377, GSE98091 and GSE109483 were analyzed with GEO2R and GREIN [32]. Expression and correlation analysis of HOTAIR and MET performed with LncTarD [33]. Expression values plotted and statistically analyzed with Prism 8.

### Zebrafish xenograft assay

Zebrafish xenograft assay was performed with SNU-449 MOCK and SNU-449 HOTAIR cell clones. The experiment was performed as described in a previous study [30]. Briefly, 48hpf embryos were anesthetized and DiI labeled cells were injected to the yolk sack.100 cells per embryo were injected, successful injections to yolk with localized cells were selected 4 h after injection. Xenografts were analyzed with Olympus SZX16 Fluorescent stereomicroscope. Three independent experiments were performed and each group consisted average number of 50 embryos with intact tumorospheres. All experiments were performed in compliance with local ethics regulations and EU Directive 2006 and approved by IBG Animal Ethics Committee with the protocol number of 03/2019.

### Statistical analysis

Statistical analysis performed using the GraphPad Prism 8 software. Statistical methods included Analysis of variance (ANOVA), Student’s t-test and linear regression. Zebrafish xenograft data was statistically analyzed with chi-square with Yates correction as described in previous study [34]. Results with *p < 0.05* were considered as statistically significant.

## Results

### Expression of HOTAIR is low in HCC cells with high c-Met expression and activation

**Fig. 1:**
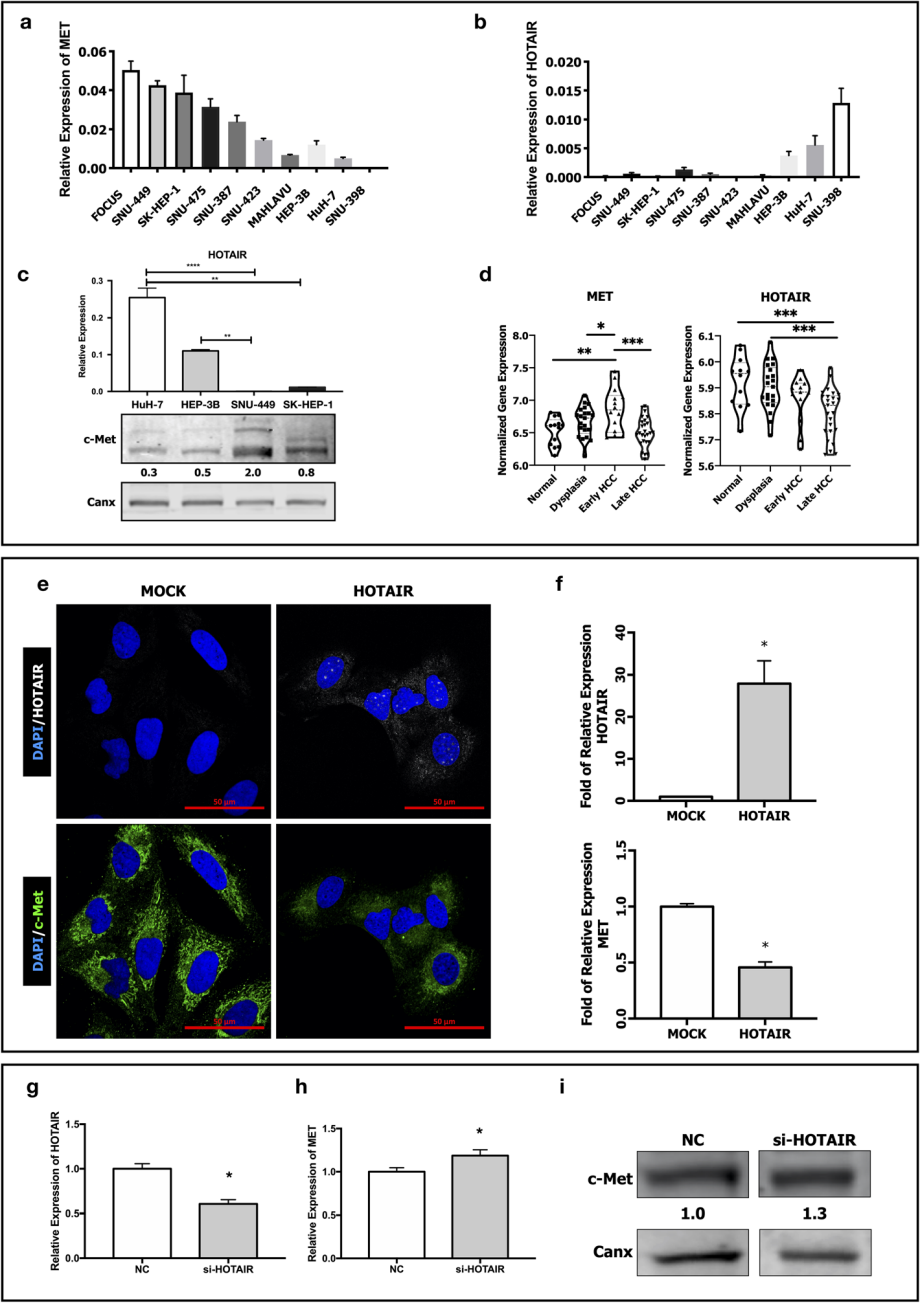
Expression of HOTAIR and c-Met in HCC cell lines and patient tissues through HCC progression. RT-qPCR analysis of (**a)** MET and (**b**) HOTAIR expressions in HCC cell lines FOCUS, SNU-449, SK-HEP-1, SNU-475, SNU-387, SNU-423, MAHLAVU, Hep-3B, HuH-7 and SNU-398. (**c**) RT-qPCR analysis of HOTAIR expression and immunoblotting of c-Met protein in HuH-7, Hep-3B, SNU-449 and SK-HEP-1 cells. (**d**) Normalized expression levels of MET and HOTAIR in normal, dysplasia, early and late HCC tissues in microarray dataset GSE89377. (**e**) Confocal microscopy image of fluorescent-in-situ hybridized HOTAIR mRNA and immunofluorescent labeled c-Met protein expression in control (MOCK) and HOTAIR over-expressing (HOTAIR OE) SNU-449 cells. RT-qPCR analysis of (**f)** MET and HOTAIR mRNA expression in MOCK and HOTAIR OE clones. RT-qPCR analysis of (**g**) HOTAIR and (**h**) c-Met mRNA expression in control si-RNA (NC) and si-HOTAIR transfected HuH-7 cells. Western blot analysis of (**i**) c-Met protein expression in control (NC) and si-HOTAIR transfected HuH-7 cells. Densitometric analysis of band intensities in immunoblots were analyzed with ImageJ and normalized with internal control protein band intensities. All graphs of experiments are presented as the mean ± SEM of at least 3 independent experiments. * p ≤ 0.05, ** p ≤ 0.01, *** p ≤ 0.001, **** p ≤ 0.0001

To examine expression levels of HOTAIR and c-Met, we analyzed their mRNA expression in 10 HCC cell lines (FOCUS, SNU-449, SK-HEP-1, SNU-475, SNU-387, SNU-423, MAHLAVU, Hep-3B, HuH-7 and SNU-398). HOTAIR expression was only abundant in HuH-7 and SNU-398 cell lines which are also known to have low or no c-Met expression, respectively (Fig. 1a-b). To evaluate the potential relation between HOTAIR and c-Met protein expressions, we selected two cell lines with constitutive c-Met activation (SNU-449 and SK-HEP-1) and two with low c-Met expression (HuH-7 and Hep-3B). RT-qPCR analysis of HOTAIR mRNA expression levels and western blot analysis of c-Met protein expression revealed that HOTAIR expression is low in HCC cell lines with high c-Met protein expression (Fig. 1c). To understand the relationship between HOTAIR and c-Met expression in HCC, we analyzed HOTAIR and MET gene expression in a microarray dataset, GSE89377, which comprise gene expression analysis in patient tissues from normal liver, dysplasia, early and late HCC stages [35]. Expression analysis of the dataset showed that HOTAIR expression decreases through HCC progression whereas MET gene expression increases (Fig. 1d).

To evaluate the effect of ectopic HOTAIR expression on c-Met expressing HCC cells, we overexpressed HOTAIR in SNU-449 cell line which has constitutive c-Met activation. Overexpression of HOTAIR (HOTAIR OE, presented in figures as HOTAIR clone) and its effect on c-Met expression were analyzed and confirmed with confocal microscopy imaging of fluorescent in-situ hybridization (FISH) of HOTAIR, immunofluorescent labeling of c-Met (Fig. 1e), and also with RT-qPCR (Fig. 1f). HOTAIR overexpression suppressed both mRNA and protein levels of c-Met, dramatically.

To understand the possible interplay between two molecules in detail, we knocked down HOTAIR expression with siRNA transfection in HuH-7 cell line and then analyzed mRNA and protein expression of c-Met. Analysis of three independent experiments showed that average of 45% knock-down of HOTAIR (Fig. 1g) induced c-Met mRNA (Fig. 1h) and protein expression (Fig. 1i), significantly. Uncut membrane images and expression levels are presented in Supplementary Fig. 1a).

### lncRNA HOTAIR overexpression suppresses c-Met downstream signaling and modulates its cellular localization via suppressing Caveolin-1 expression

**Fig. 2:**
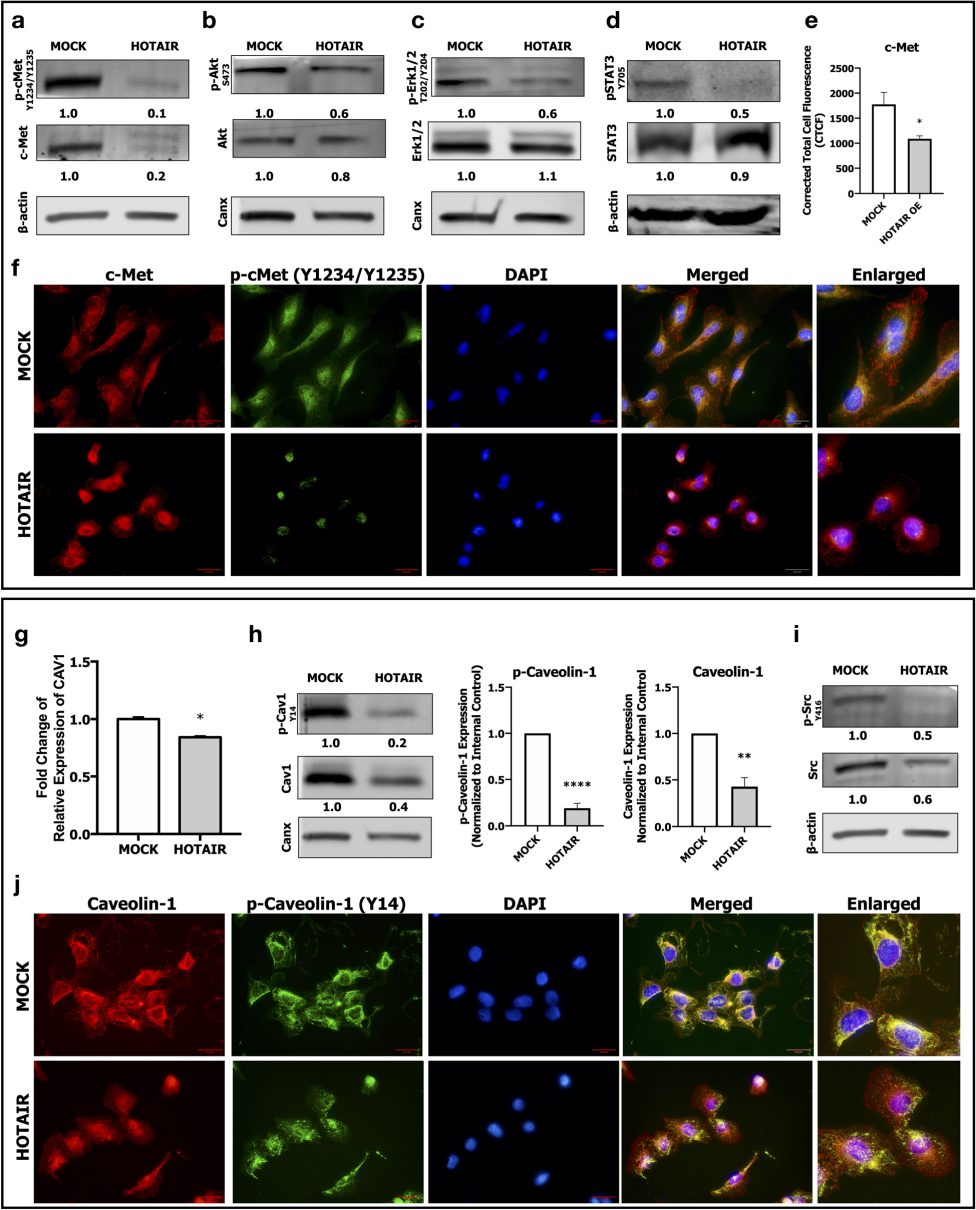
Effect of HOTAIR overexpression in c-Met downstream signaling. Analysis of c-Met and downstream signaling in MOCK and HOTAIR OE cells. Immunoblot analysis of (**a**) c-Met protein expression, c-Met activation phosphorylation (Tyr-1234/Tyr-1235); (**b**) Akt1 protein expression, Akt1 activation phosphorylation (Ser-473); (**c**) Erk1/2 protein expression, Erk1/2 activation phosphorylation (Thr-202/Tyr-204); (**d**) STAT3 protein expression and activation phosphorylation (Tyr-705). Column graph of corrected total cell fluorescence of c-Met immunofluorescent staining (**e**). Immunofluorescence imaging of (**f**) total c-Met protein (red) and its activation (green). RT- qPCR analysis of (**g**) CAV1 mRNA expression, immunoblotting of (**h**) Caveolin-1 protein and its activation phosphorylation (Tyr-14) and column graph of densitometric analysis of Caveolin-1 activation and total protein expressions. Immunoblotting of (**i**) Src kinase expression and its activation phosphorylation (Tyr-416). Immunofluorescence imaging of (**j**) total Caveolin-1 protein (red) and its activation (green). DAPI (blue) was used as a nuclear marker in immunofluorescent images. Densitometric analysis of band intensities in immunoblots were analyzed with ImageJ and normalized with internal control protein band intensities. All graphs of experiments are presented as the mean ± SEM of at least 3 independent experiments. * p ≤ 0.05, ** p ≤ 0.01, *** p ≤ 0.001, **** p ≤ 0.0001

To understand the effects of HOTAIR on c-Met signaling, we analyzed activation of c-Met and its downstream signaling pathways Akt, MAPK and STAT3. Western blot analysis showed that HOTAIR OE suppressed c-Met protein expression and activation (Fig. 2a). Repression of c-Met activation was a result of diminished c-Met protein expression and there was not a significant inhibition on c-Met phosphorylation (p-cMet) when normalized to total c-Met protein expression (Supplementary Fig. 1b). HOTAIR OE caused a modest decrease in Akt activation (Fig. 2b), and suppressed MAPK (Fig. 2c) and STAT3 (Fig. 2d) activations, dramatically. We confirmed these results by analyzing expression and activation of c-Met by immunofluorescent microscopy (Fig. 2e and f). Macroscopic analysis of the cells revealed that HOTAIR OE cells were smaller with roundish morphology and even the immunofluorescence staining intensities of c-Met seem to be higher in HOTAIR OE cells, analysis of immunofluorescence intensity per cells showed that is an optical illusion caused by size (smaller) and morphological (rounder) attributes of HOTAIR OE cells (Fig. 2e). Furthermore, immunofluorescent labeling revealed a differential organization and localization of c-Met in HOTAIR OE cells (Fig. 2f). In our previous studies, we reported that Caveolin-1 (CAV1), a membrane protein enriched in lipid-rafts, enhances c-Met activation and modulates its membrane localization in HCC cells [27]. To investigate the possible role of CAV1 in HOTAIR/c-Met interaction, we analyzed expression and activation of CAV1 and its activating kinase, Src, in HOTAIR OE cells. HOTAIR OE suppressed CAV1 mRNA level (Fig. 2g), protein expression and activation (Fig. 2, Supplementary Fig. 1c) as well as Src kinase expression and activation (Fig. 2i). Moreover, immunofluorescent staining revealed that membrane organization of Caveolin-1 was disrupted in HOTAIR OE cells which was compatible with disorganization of c-Met (Fig. 2j).

### Overexpression and/or activation of c-Met decreases HOTAIR expression in HCC cell lines

**Fig. 3:**
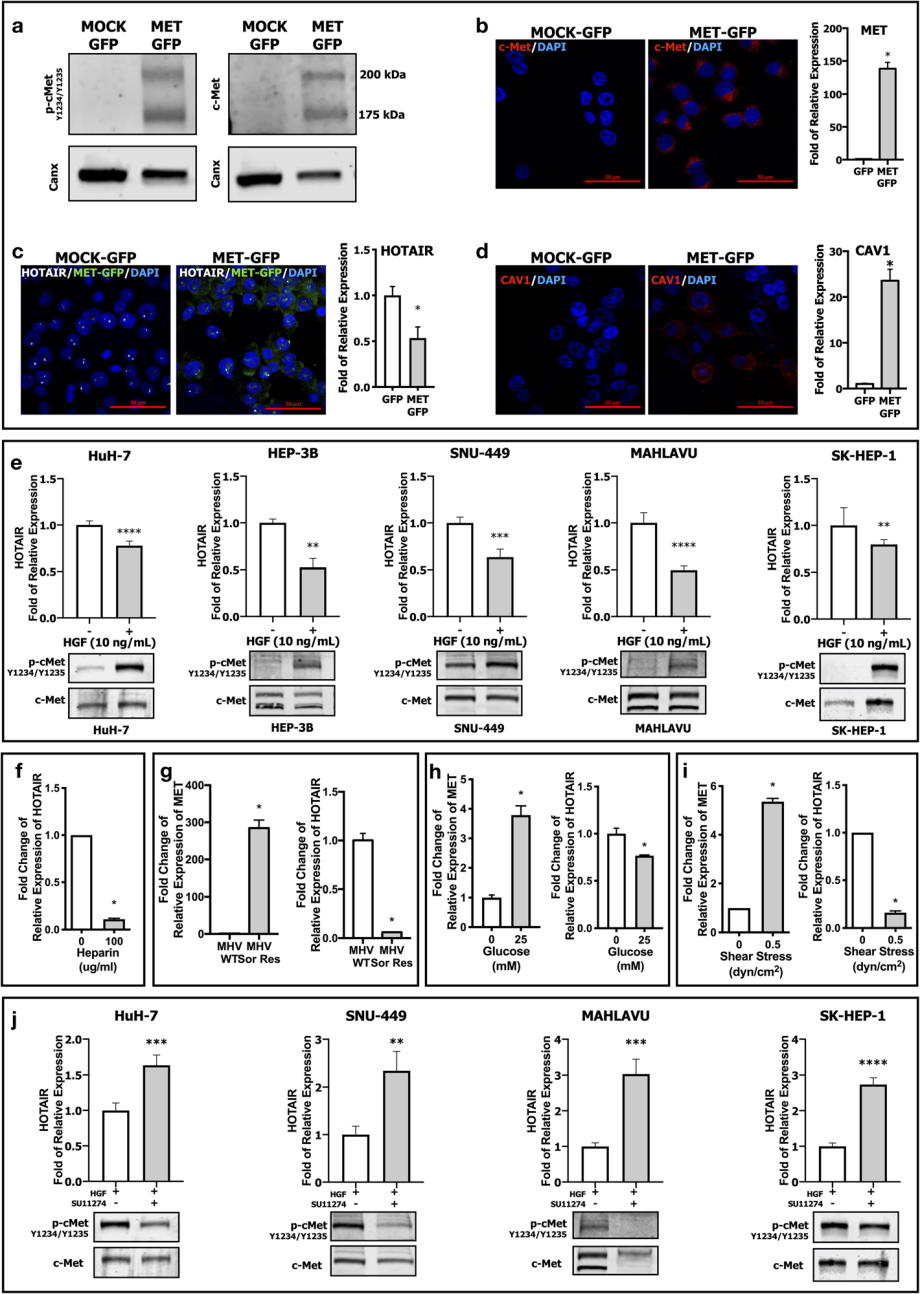
Analysis of HOTAIR-c-Met interaction in MET overexpressed, activated and inhibited conditions. Immunoblotting of (**a**) c-Met activation phosphorylation (Tyr-1234/Tyr-1235), and total c-Met protein expression. Confocal microscopy imaging of (**b**) c-Met protein and RT-qPCR analysis of c-Met mRNA expression in MET OE SNU-398 clones. (**c**) Confocal imaging of FISH labeling (white dots) and RT-qPCR analysis of HOTAIR mRNA expression in MET OE clones. (**d**) Confocal imaging of Caveolin-1 protein and qPCR analysis of CAV1 mRNA expression in MET OE cells. (**e**) Immunoblot of c-Met activation and qPCR analysis of HOTAIR expression in ligand-dependent activation (HGF 10 ng/ml) of c-Met in HuH-7, HEP-3B, SNU-449, MAHLAVU and SK-HEP-1 cells. RT-qPCR analysis of HOTAIR expression (**f**), in response to heparin (100 μg/mL) induced c-Met activation in SK-HEP-1 cells. RT-qPCR analysis of MET and HOTAIR expressions in sorafenib resistance (MAHLAVU cell line) (**g**) and high-glucose (25 mM, HuH-7 cell line) (**h**) and fluidic shear stress (0.5 dyn/cm^2^, HuH-7 cell line) (**i**) induced c-Met activations. (**j**) Immunoblot of c-Met activation and qPCR analysis of HOTAIR expression in tyrosine kinase activity inhibition of c-Met by SU11274 (2500 nM) HuH-7, SNU-449, MAHLAVU and SK-HEP-1 cells. All graphs of experiments are presented as the mean ± SEM of at least 3 independent experiments. * p ≤ 0.05, ** p ≤ 0.01, *** p ≤ 0.001, **** p ≤ 0.0001

To understand the interplay between HOTAIR and c-Met further, we applied an opposite approach by overexpressing c-Met in SNU-398 HCC cell line which lacks c-Met protein expression and activation in wild-type (Fig. 1a). Overexpression and activation phosphorylation of c-Met were confirmed by western blot (Fig. 3a), immunofluorescent labeling and RT-qPCR analysis (Fig. 3b). Analysis of HOTAIR expression in MET-OE-SNU 398 (presented in figures as MET-GFP) cells by FISH and RT-qPCR showed that HOTAIR mRNA expression was significantly suppressed by c-Met overexpression (Fig. 3c). To investigate HOTAIR/c-Met/Caveolin-1 axis further, we analyzed Caveolin-1 expression in c-MET OE cell lines and found that Caveolin-1 expression was significantly upregulated, consistent with HOTAIR down-regulation (Fig. 3d).

To understand the role of c-Met activation in HOTAIR expression, we analyzed the effect of HGF induced c-Met activation on HCC cell lines with different c-Met and HOTAIR expression levels such as HuH-7, HEP-3B, SNU-449, MAHLAVU and SK-HEP-1. Ligand-dependent c-Met activation by 10 ng/ml HGF for 1 h suppressed HOTAIR expression significantly in all tested cell lines (Fig. 3e). In addition to ligand-dependent activation, we examined the role of ligand-independent c-Met activation on HOTAIR transcription. In our previous studies, we showed that heparin treatment [36], acquired sorafenib resistance [5], high glucose induction [37] or fluidic shear stress treatment [38] induces ligand independent c-Met activation in HCC cell lines. Similar to HGF, heparin (Fig. 3f), sorafenib (Fig. 3g), high-glucose (Fig. 3h) and fluidic shear stress (Fig. 3i) treatments induced c-Met activation resulted in a substantial decrease in HOTAIR transcription. The maintenance of the interplay between HOTAIR and c-Met in different contexts supports the hypothesis that there is a reciprocal crosstalk between these two molecules in HCC cell lines. We further confirmed the rescue of HGF-suppressed HOTAIR expression after c-Met tyrosine kinase inhibition by SU11274 in HCC cell lines HuH-7, SNU-449, MAHLAVU and SK-HEP-1. c-Met tyrosine kinase inhibition rescued HGF-induced HOTAIR suppression in HCC cell lines (Fig. 3j).

To understand the biological significance of this interaction, we investigated the phenotypic and biological results of HOTAIR overexpression in SNU-449 HCC cell line.

### HOTAIR OE induced c-Met inhibition decreases adhesion, proliferation and colony formation of SNU-449 cells

**Fig. 4:**
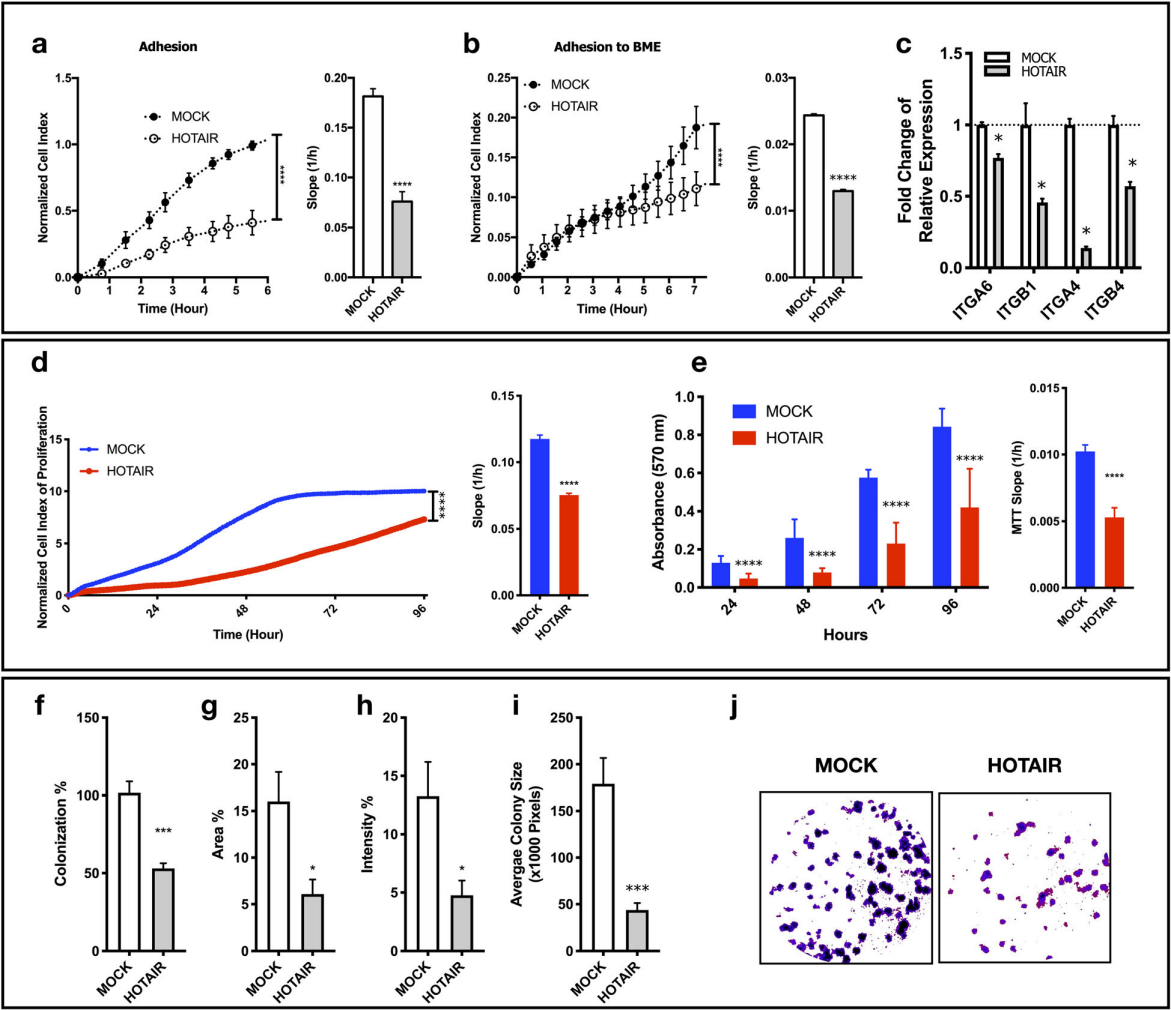
HOTAIR OE induced c-Met inhibition decreases adhesion, proliferation and colony formation. Normalized cell index and slope analysis of adhesion to (**a**) cell culture treated and (**b**) basal membrane extract-coated surfaces analyzed with xCELLigence RTCA. (**c**) RT-qPCR analysis of ITGA6, ITGB1, ITGA4 and ITGB4 expressions. (**d**) Analysis of normalized cell index and rate (slope) of proliferation by xCELLigence RTCA system. Normalized (**e**) MTT absorbance (570 nm) and proliferation rate (slope) calculated by MTT absorbance. 2D colony formation analysis of MOCK and HOTAIR clones presented as (**f**) percentage of colonization, (**g**) percentage of area covered by colonies, (**h**) intensity of Giemsa-staining (**i)** average colony size in pixels and (**j**) representative images of clones generated with ImageJ plugin ColonyArea. 2D colony formation is analyzed by ImageJ plugin ColonyArea. All graphs of experiments are presented as the mean ± SEM of at least 3 independent experiments. * p ≤ 0.05, ** p ≤ 0.01, *** p ≤ 0.001, **** p ≤ 0.0001

HOTAIR OE cells showed reduced adhesion capacity to cell culture-treated (Fig. 4a) and basal membrane extract coated cell culture surfaces (Fig. 4b) in xCELLigence real time cell analysis system (RTCA). To understand the cause of decreased extracellular matrix (ECM) attachment capacity, we analyzed the expression of cell surface integrins that contribute to matrix attachment. ITGA6, ITGB1, ITGA4 and ITGB4 expressions were down-regulated in HOTAIR OE cells, significantly (Fig. 4c).

Proliferation of the cells were evaluated with both xCELLigence RTCA system (Fig. 4d) and MTT analysis (Fig. 4e). HOTAIR OE cells showed significant reduction in proliferation rate in both techniques. Compatible with proliferation assays, 2D colony forming potential of HOTAIR OE cells were dramatically low. Colony counts are normalized to number of cells plated for evaluation of colonization percentage. HOTAIR OE cells showed significantly lower colonization ability in comparison to MOCK cells (Fig. 4f). Coverage area of colonies in well-plates, staining intensity of colonies and average colony sizes were also significantly lower in HOTAIR OE cells (Fig. 4g-j). These results reflect lower capacities of adhesion and proliferation.

### HOTAIR OE induced c-Met inhibition decreases motility and invasion capacity of SNU-449 cells

**Fig. 5:**
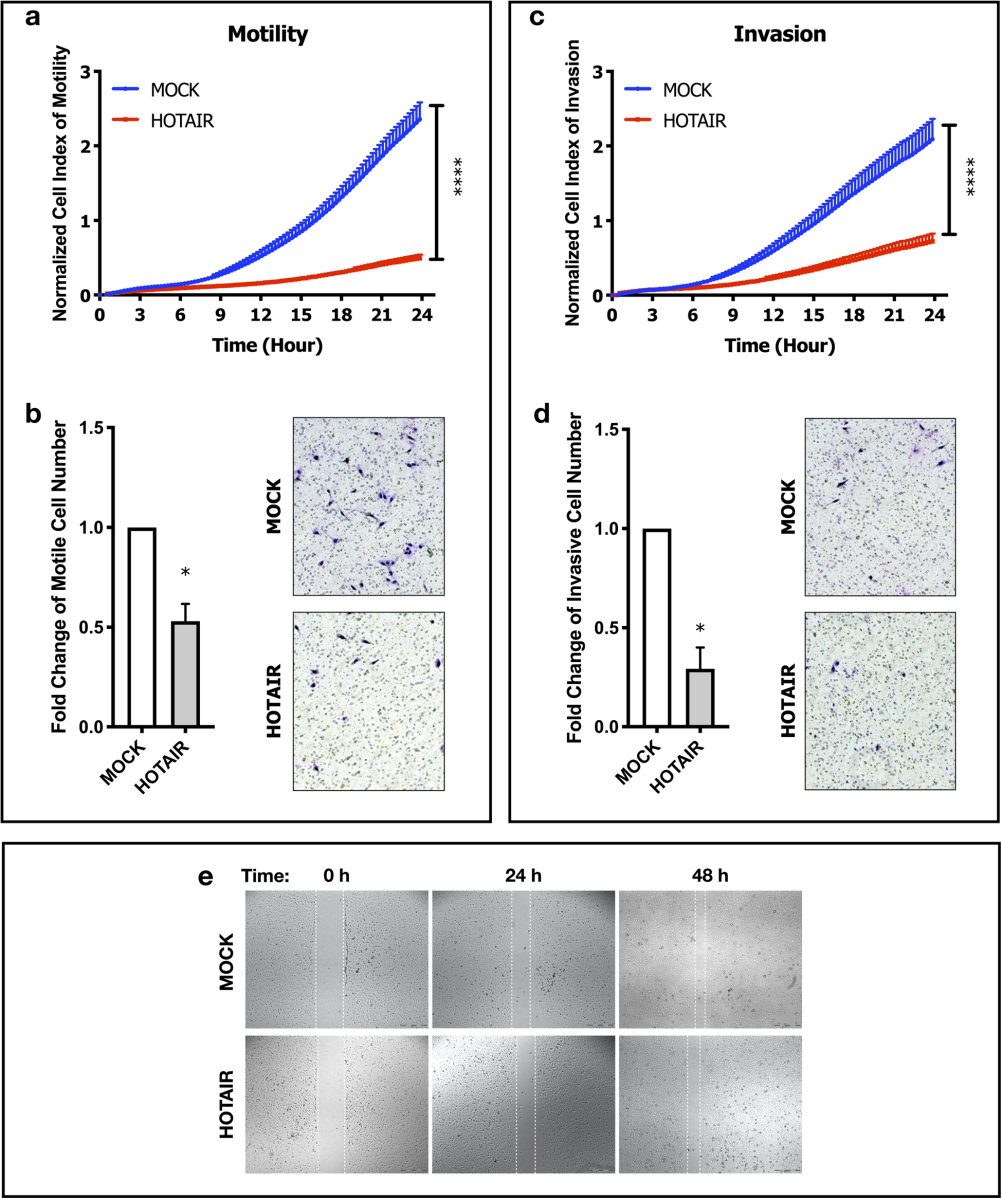
HOTAIR OE induced c-Met inhibition decreases individual motility and invasion ability of SNU-449 cells. Real-time (by xCELLigence RTCA) and end-point (cell culture inserts) analysis of motility and invasion of MOCK and HOTAIR OE cell clones. Normalized cell index of (**a**) motility and (**c**) invasion analyzed by xCELLigence RTCA system. Fold change graphs and brightfield images of (**b**) motile and (**d**) invaded cells in trans-well cell culture inserts. (**e**) Brightfield microscopy images of 0-, 24- and 48-h scratch (wound-healing) assay of MOCK and HOTAIR OE clones. All graphs of experiments are presented as the mean ± SE of at least 3 independent experiments. * p ≤ 0.05, **p ≤ 0.01, ***p ≤ 0.001

Motility and invasion experiments were performed in parallel by both xCELLigence RTCA system and trans-well cell culture systems. Cell index of motile (Fig. 5a) and invasive (Fig. 5c) cells in HOTAIR OE clones were significantly lower in xCELLigence RTCA (p ≤ 0.001). Consistent with real-time motility and invasion analysis; the number of motile and invasive cells migrated through trans-well cell culture inserts were reduced in HOTAIR OE cells (Fig. 5b, d), significantly (p ≤ 0.05).

Since trans-well systems especially tests individual cell movement ability, we further analyzed collective migration ability of clones in scratch (wound-healing) assay. Scratch assay enables cells to move collectively, without losing cell-cell attachments. Even though HOTAIR OE cells have reduced ability to migrate individually, they were still motile. Rather than migrating individually, HOTAIR OE cells migrated without losing cell-cell contacts, collectively (Fig. 5e).

### lncRNA HOTAIR inhibits c-Met induced cell scattering and promotes metastasis in zebrafish embryos

**Fig. 6:**
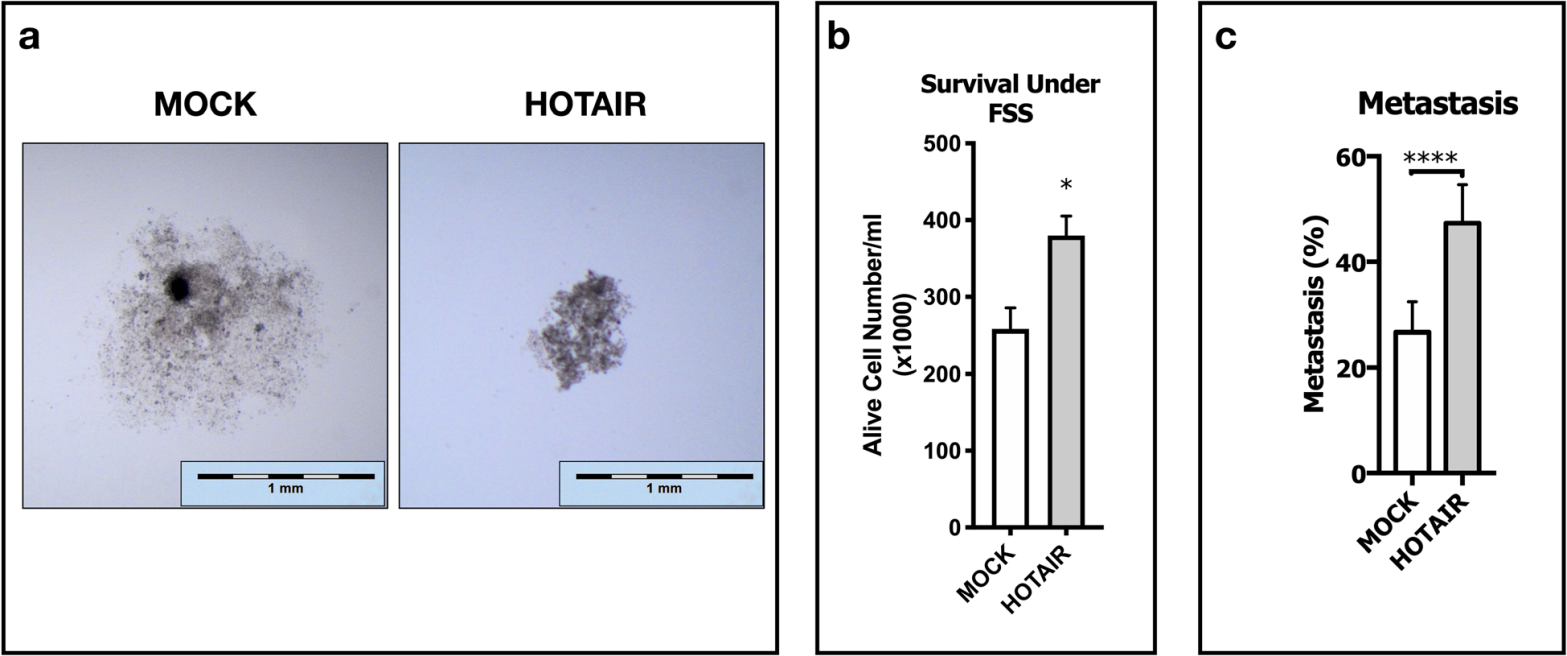
lncRNA HOTAIR suppresses c-Met induced cell scattering, enhances survival under FSS and promotes metastasis in zebrafish embryos. Spheroid formation, survival under fluidic shear stress and metastatic capacity of MOCK and HOTAIR OE cells. (**a**) Spheroid images were collected with stereo-microscopy and magnified equally with scale-bars to generate more clarified presentation of data. (**b**) Graphical presentation of alive cell numbers of MOCK and HOTAIR OE clones cultured under 0.5 dyn/cm^2^ fluidic shear stress for 1 h. Embryonic zebrafish xenograft experiments with MOCK and HOTAIR OE SNU-449 cell clones presented as (**c**) percentage of metastasis in groups that were analyzed in total of three biological experiments. Statistical significance was analyzed by chi-square with Yates correction. All graphs of experiments are presented as the mean ± SE of at least 3 independent experiments. * p ≤ 0.05, ** p ≤ 0.01, *** p ≤ 0.001, **** p ≤ 0.0001

Decrease in attachment capacity to ECM led us to investigate spheroid formation capacity of HOTAIR OE cells. We analyzed spheroid formation ability of those cells with hanging-drop spheroid formation assay. SNU-449 cells have a complete mesenchymal phenotype, as a result of scattering effect of constitutive c-Met activation, and they are not inclined to form spheroids in hanging-drops. Due to their reduced ability to form cell-cell contacts maintained by constitutive c-Met activation; MOCK cells formed loose, scattered and wide clusters whereas HOTAIR OE cells formed more tight, stacked and small spheroids (Fig. 6a). Significant ability to form intact spheroids led us to investigate their survival capacity under fluidic shear stress (FSS). To test their survival ability, cells sheared for 1 h under FSS of 0.5 dyn/cm^2^, mimicking the sinusoidal shear stress in liver [39]. HOTAIR OE improved survival ability under sinusoidal FSS. Alive cell numbers after one hour of FSS was significantly higher (p ≤ 0.05) in HOTAIR OE clones (Fig. 6b).

Finally, embryonic zebrafish xenograft model was used to test in vivo metastatic capacity of HOTAIR OE, further. Average number of 100 HOTAIR OE and MOCK cells were injected to the yolk sac of 48 h zebrafish embryos and embryos which bear an intact tumorosphere after 4 h of injection were raised for 4 days to analyze metastatic capacity. Three independent experiments were performed and in every experiment each group consisted an average number of 50 embryos with intact tumorospheres. Metastatic capacity was calculated by number of xenograft hosts that have metastasized cells at 4 days post injection (dpi). Even though wild-type SNU-449 cells are known to be metastatic in embryonic zebrafish model [30], HOTAIR OE dramatically increased their metastatic capacity when compared to MOCK cells (Fig. 6c).

### lncRNA HOTAIR maintains hybrid E/M phenotype to promote metastasis

**Fig. 7:**
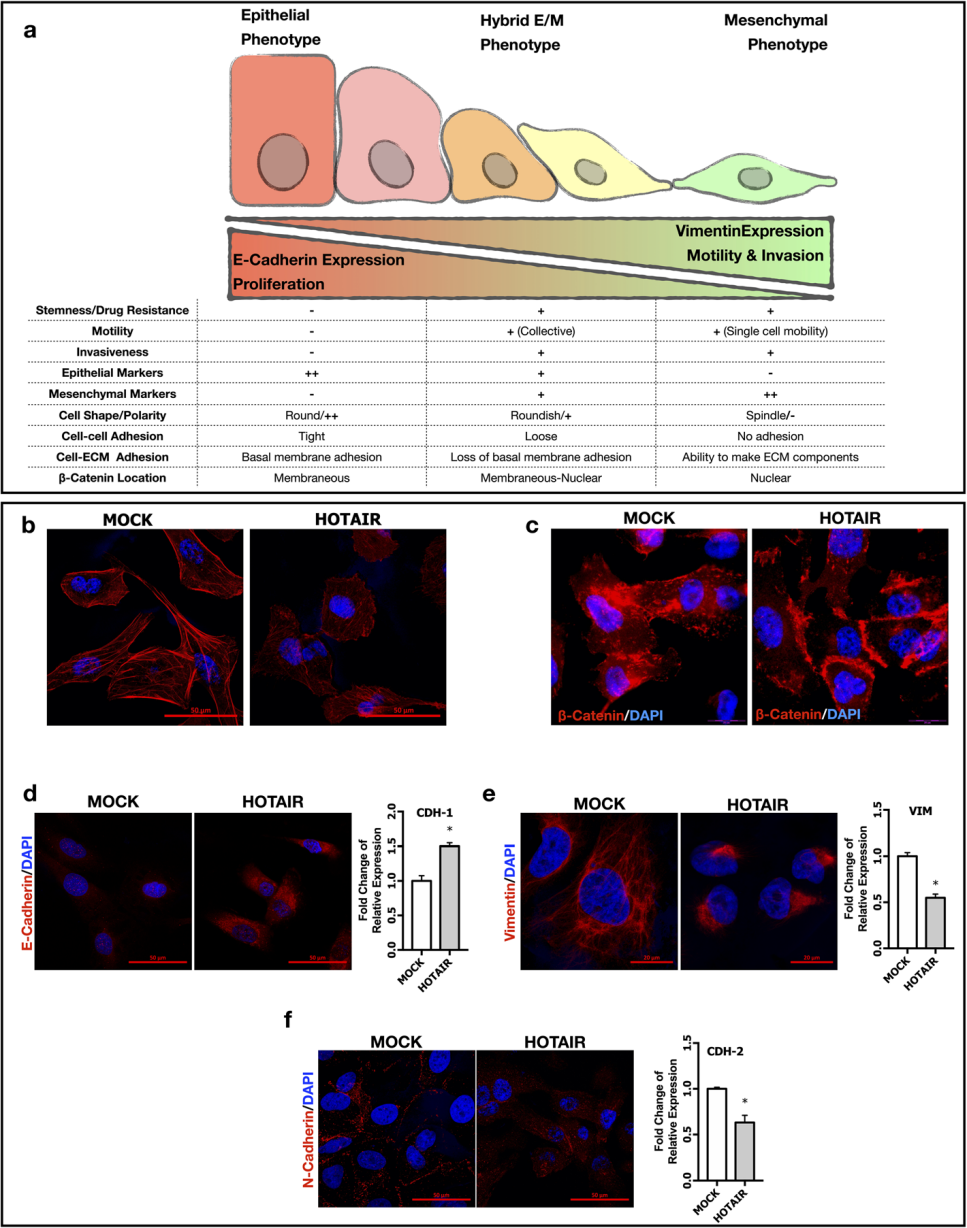
lncRNA HOTAIR maintains hybrid E/M phenotype to promote metastasis. (**a**) Schematic presentation of morphological, molecular and biological biomarkers of epithelial, mesenchymal and hybrid E/M phenotypes. Confocal imaging of (**b**) Alexa-555 labeled phalloidin staining of F-actin filaments (red). Immunofluorescence imaging of (**c**) beta-Catenin protein (red) and its cellular localization. Confocal imaging and RT-qPCR analysis of (**d**) E-Cadherin (**e**) Vimentin and (**f**) N-Cadherin expressions. Alexa-594 conjugated secondary antibodies were used in all immunolabeling and confocal imaging experiments. DAPI (blue) was used as a nuclear marker in immunofluorescence and confocal images. All graphs of experiments are presented as the mean ± SEM of at least 3 independent experiments. * p ≤ 0.05

Our experiments with HOTAIR OE cells show morphological and biological properties that are consistent with the hybrid E/M phenotype which can be defined as a fluidic state between epithelial and mesenchymal phenotypes (Fig. 7a). To test this hypothesis, we examined previously defined EMT markers in HOTAIR OE SNU-449 cells [40–42]. First, we found that in contrast to the mesenchymal and elongated phenotype, HOTAIR OE cells were round-shaped with thinner F-actin stress fibrils indicated with phalloidin staining (Fig. 7b). Then, we performed a detailed investigation of molecular biomarkers of hybrid E/M phenotype. In HOTAIR OE cells, beta-catenin was enriched in the plasma membrane (Fig. 7c), expression of epithelial biomarker E-cadherin was upregulated (Fig. 7d) whereas mesenchymal biomarker Vimentin expression was significantly down-regulated and showed polarity (Fig. 7e). Consistent with epithelial phenotype, N-Cadherin expression was down-regulated and mostly located in the plasma membrane of HOTAIR OE cells (Fig. 7f). Immunofluorescent analysis of cytoskeletal organization of F-actin and Vimentin in SNU-398 MET OE clones showed that ectopic MET expression and its suppressive effect on HOTAIR expression was also resulted in thickening of F-actin stress fibrils, and increase in Vimentin expression and polarity (Supplementary Fig. 1d).

**Fig. 8:**
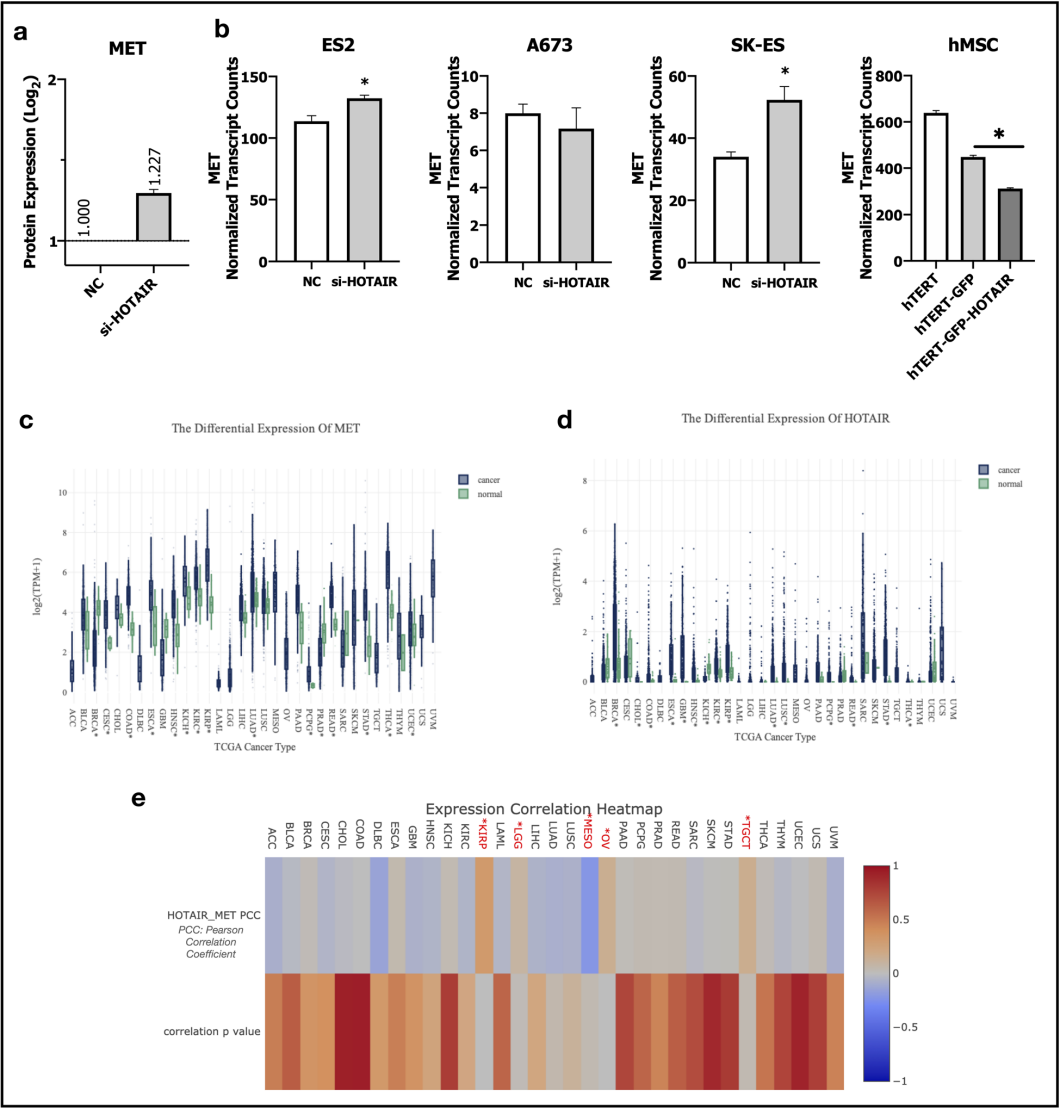
Bioinformatic analyses of GEO and TCGA datasets supports HOTAIR and c-Met interplay. (**a**) Column graph of c-Met protein expression in control-siRNA and HOTAIR-siRNA transfected HepG2 cells in GEO dataset GSE98091. (**b**) MET gene expression in experimental groups of GEO dataset GSE109483. Column graphs of control-GapMeR and HOTAIR-GapmeR transfected Ewing sarcoma cell lines ES2, A673 and SK-ES (1st, 2nd and 3rd graphs). Last graph belongs to hTERT-immortalized human mesenchymal stem cells (hMSC), hMSCs transfected with GFP (hTERT-GFP) and HOTAIR-GFP (hTERT-GFP-HOTAIR) overexpression vectors. Analysis of (**c**) MET (**d**) HOTAIR gene expressions in TCGA datasets. (**e**) Heatmap of gene expression correlation analysis of HOTAIR and MET in TCGA datasets. GEO dataset analysis are performed with GREIN and TCGA gene expression and correlation analysis are performed with LncTarD. * p ≤ 0.05

In addition to in-vitro experiments, we performed bioinformatic analysis of available data-sets GSE98091 and GSE109483. GSE98091 dataset has an integrated RNA sequencing transcriptomic and quantitative proteomic analysis those performed to explore the regulatory role of HOTAIR in HCC [43]. Researchers inhibited HOTAIR expression by siRNA silencing in HepG2 cells and analyzed differentially expressed genes and proteins between control-siRNA and HOTAIR-siRNA HepG2 clones. Even though transcriptional dysregulation of MET was not significant in this study, protein expression of c-Met was significantly higher in HepG2 cells transfected with HOTAIR siRNA (Fig. 8a). In GSE109483 dataset, researchers repressed HOTAIR expression in three Ewing sarcoma cell lines (ES2, A673, SK-ES) and overexpressed HOTAIR in hTERT-immortalized human mesenchymal stem cells to evaluate its effects on gene expression [44]. They performed RNA-Seq in Ewing sarcoma cell lines (control-GapmeR and HOTAIR repressing GapmeR clones), and hTERT-immortalized hMSCs with expression of control GFP and HOTAIR. MET gene expression was significantly upregulated by HOTAIR repression in ES2 and SK-ES Ewing Sarcoma cell lines and its expression was significantly repressed by HOTAIR over-expression in hTERT-immortalized human mesenchymal stem cells (Fig. 8b). The data of these datasets supports proposed interaction of HOTAIR and MET and they are very important to show similar results with HCC in different cancer and cellular contexts.

Finally, we analyzed TCGA datasets of various cancers to have a foresight about the possible relationship of two molecules [3]. HOTAIR and MET gene transcripts in TCGA datasets were analyzed by GREIN and LncTarD [32, 33]. Despite the limitations in RNAseq sample counts among TCGA datasets that comprise RNA sequencing of long non-coding RNAs, HOTAIR and c-Met were differentially expressed between normal and primary tumor tissues in various cancer types (Fig. 8c-d). While MET expression was significantly suppressed in primary tumor tissues in breast invasive carcinoma (BRCA) and upregulated in kidney chromophobe (KIHC), HOTAIR expression was significantly higher and lower, respectively (Fig. 8c-d). Analysis of the correlation of these two molecules among TCGA datasets showed that and they were reversely correlated with each other in mesothelioma (MESO) but positively correlated in kidney renal papillary cell carcinoma (KIRP), brain lower grade glioma (LGG), ovarian serous cystadenocarcinoma (OV) and testicular germ cell tumors (TGCT) with statistical significance. Even lack of significance, tendency of reverse regulation was significance in adrenocortical carcinoma (ACC), cervical squamous cell carcinoma and endocervical adenocarcinoma (CESC), lymphoid neoplasm diffuse large b-cell lymphoma (DLBC), glioblastoma multiforme (GBM), head and neck squamous cell carcinoma (HNSC), kidney renal clear cell carcinoma (KIRC), acute myeloid leukemia (LAML), liver hepatocellular carcinoma (LIHC), lung adenocarcinoma (LUAD) and ulveal melanoma (UVM), (Fig. 8e). Although lncRNA HOTAIR, one of the most studied long non-coding RNAs, is reported to be elevated in patients with metastasis and poor prognosis in many cancer types, analysis of HOTAIR in TCGA datasets revealed that HOTAIR has a context-dependent expression pattern which is not always upregulated through the progression of the disease (Fig. 8d-e).

## Discussion

Our results suggest c-Met and HOTAIR axis as a modulator of epithelial/mesenchymal hybrid state in hepatocellular carcinoma cells. Mechanistically, we demonstrated that the interplay between c-Met receptor tyrosine kinase and HOTAIR is critical for maintenance of hybrid E/M state in HCC cells to cope with rate-limiting steps of tumor metastasis, such as survival in adhesion independent microenvironment, escaping from anoikis and resisting to fluidic shear stress (FSS) in HCC.

HCC cell lines with high c-Met expression and activation are defined to have mesenchymal phenotype in previous definitive studies [45, 46]. In our study, we report that HCC cells can be divided into two clusters which show differential expression tendency for HOTAIR and c-MET genes. Expression of these two molecules among 10 HCC cell lines demonstrated that most HCC cell lines show a reverse tendency in levels of c-Met and HOTAIR expression. To further confirm the possible reverse interaction between HOTAIR and c-Met, we overexpressed HOTAIR in SNU-449 cell line which has ligand-independent constitutive c-Met activation and silenced HOTAIR in HuH-7 cell line which expresses HOTAIR, abundantly. Our results show that constitutive c-Met expression is suppressed by HOTAIR over-expression and HOTAIR silencing induced c-Met expression in HCC cells. It is very important to note that inhibition of expression with siRNAs is limited mostly with cytoplasmic HOTAIR mRNA targeting and further studies should be done to address the detailed mechanism of HOTAIR action in means of molecular interactions and/or epigenetic function in HCC cells.

Analysis of microarray dataset comprised of tissues from normal liver, dysplastic liver, early and late HCC of patients (GSE89377) showed that HOTAIR was downregulated through hepatocarcinogenesis whereas c-Met expression was upregulated. This analysis supports our data showing HOTAIR downregulation is a requirement for c-Met activation and c-Met triggered complete mesenchymal phenotype in HCC cell lines. Contrary to the reports of melanoma, retinoblastoma and colon cancer studies [47–49] and expectations on the parallel expression tendencies of c-Met and HOTAIR in HCC cell lines; comparative analysis of HCC cell lines and cancer datasets shows the opposite, clearly. Indicated studies suggested HOTAIR as a competing long non-coding RNA decoying microRNAs targeting c-Met in different cancer contexts, and those microRNAs are reported to have different molecular targets in HCC rather than c-Met [50, 51].

In addition to suppression of c-Met protein expression and activation as a consequence, HOTAIR weakens downstream Akt1, MAPK and STAT3 signaling pathways not only by decreasing c-Met expression but also by modulating its membrane organization. In our previous studies, we showed that Caveolin-1 enhances c-Met signaling by co-localizing in plasma membrane [27]. Src kinase, a well-identified downstream effector of c-Met, phosphorylates Caveolin-1 from Tyr-14 residue to induce lipid-raft enrichment of c-Met with Caveolin-1 and formation of caveola structures [27, 52]. HOTAIR over-expression reduced Caveolin-1 expression and activation via suppressing activation of c-Met and its downstream effector Src kinase. In parallel to c-Met, Caveolin-1 organization in plasma membrane was also disrupted in HOTAIR OE cells. Tsai et al. defined function of HOTAIR in epigenetic regulation of its target genes and they reported Caveolin-1 as one of the most differentially expressed genes in response to HOTAIR knock-down in foreskin fibroblasts [12]. In addition to the HOTAIR OE data, we showed that HOTAIR *knock-down* elevated CAV1 expression in HuH-7 cells (Supplementary Fig. 1e). We confirmed the interaction axis of c-Met, HOTAIR and Caveolin-1 is conserved in c-Met over-expression model of SNU-398 cell line. Its wild type is lack of c-Met expression but expresses HOTAIR, abundantly. In addition to the lack of c-Met expression, SNU-398 cells also do not express Caveolin-1 [26]. As a consequence of c-Met overexpression and HOTAIR suppression, Caveolin-1 expression was induced in those cells. In addition to the over-expression studies of c-Met and HOTAIR, we showed that c-Met and HOTAIR reverse interaction is conserved in both ligand-dependent and independent c-Met activation contexts and also c-Met kinase inhibition. These results support the defined interplay between two molecules.

To understand the biological significance of HOTAIR/c-Met interaction, we analyzed the results of HOTAIR overexpression on c-Met related biological responses which are clearly defined in the literature as contributing to aggressive phenotype of HCC cells [53]. HOTAIR over-expressing cells showed delayed adhesion to surfaces and suppressed expression of adhesion-related integrins [54]. In addition to decreased attachment to surface, proliferation and colony formation ability of HOTAIR OE cells were also decreased. Suppression of individual motility and invasion of HOTAIR OE cells were consistent with suppressed scattering effect of c-Met signaling. HOTAIR OE cells were migrating collectively rather than individually which is compatible with previous studies explaining migration behavior of cells with hybrid E/M phenotype [15]. Increased expression of cell adhesion molecules such as CDH-1 and JAM-2 (Supplementary Fig. 1f) were consistent with the evidence of collective migration that requires preservation of cell-to-cell interactions [55, 56].

Surprisingly, HOTAIR over-expressing cells showed increased survival ability in adhesion independent culture and under FSS. Escaping from anoikis and overcoming damage incurred by shear forces are important necessities of survival in circulation to achieve metastasis [57]. Response to fluidic shear-stress or adhesion-independent conditions are context-dependent processes those depend on the molecular pool of the cell; HOTAIR over-expressing cells resisted those unfavorable conditions via formation of spheres [39]. Aggregation into spheroids to overcome the stress of unfavorable microenvironment, enhancing cell-cell adhesion and surviving under damage of shear forces are well-defined requirements of metastasis [39, 57]. As expected, by attainment of those abilities, HOTAIR over-expression increased metastatic ability of SNU-449 cells which was defined to be highly metastatic in our previous studies in zebrafish xenograft model [30]. Our study is the first in the literature defining the molecular basis of HOTAIR overexpression mediated increase in the metastatic ability of HCC cells.

Since the definition of EMT has been changed and broadened, transitional states between epithelial and mesenchymal phenotypes are called “partial EMT”. Existence of intermediate hybrid phenotypes have been defined in different molecular and biological processes such as fibrosis, development, wound healing and cancer [15]. In our study, we found that HOTAIR acts as a fine tuner of maintenance of hybrid E/M phenotype by modulating c-Met signaling. Compatibly with the literature, we analyzed defined morphological and molecular markers of epithelial and mesenchymal phenotypes in HOTAIR over-expressing cells [15, 16, 41, 42]. HOTAIR OE cells were round-shaped and had thinner F-actin stress fibrils. Beta-Catenin was enriched in membrane compatible with increased E-cadherin expression and hybrid E/M phenotype characteristics [18]. In comparison with MOCK cells, mesenchymal biomarkers Vimentin and N-cadherin expression was suppressed in HOTAIR OE cells (Fig. 7). Consistent with HOTAIR over-expression data in SNU-449 cells, c-Met overexpression in SNU-398 cells increased F-actin stress fibrils and Vimentin expression and, HOTAIR *knock-down* induced Vimentin expression in HuH-7 cells (Supplementary Fig. 1 g). While over-expression of c-Met in SNU-398 cells suppressed HOTAIR expression and led acquirement of mesenchymal attributes; inhibition of HOTAIR expression in HuH-7 cells enhanced mesenchymal phenotype. Changes in cytoskeletal components and dis−/re-assembling of cell-cell contacts regulates gene expression, motility and cell-cycle. Cells placed in spectrum between epithelial and mesenchymal phenotypes are reported to arrest cell cycle and decrease their proliferative activity [15, 17]. Consistent with the literature, proliferation rate of HOTAIR OE cells were decreased (Fig. 4) and cell cycle was arrested (Supplementary Fig. 1 h-i) in these cells.

Decrease in biological responses such as individual cell motility and invasion are consistent with suppressed scattering effect of c-Met signaling activity. Even individual cell motility was decreased, HOTAIR OE clones have ability to enclose the scratch via collective migration as fast as MOCK clones in wound-healing assay (Fig. 5). Collective migration is not only important for maintenance of invading front of tumors but also it is an emerging mechanism for seeding of secondary tumors [58]. Taken together, our data shows that decrease in HOTAIR expression and its effects on c-Met and Caveolin-1 activation is crucial through the completion of mesenchymal phenotype.

Concordant with our data, we report that c-Met and HOTAIR expression profiles show inverse correlation in mesothelioma but positively correlated in kidney renal papillary cell carcinoma, brain lower grade glioma, ovarian serous cystadenocarcinoma and testicular germ cell tumors with statistical significance. With the fact that all samples in TCGA cancer datasets do not compromise long non-coding gene expression data, analysis of expression correlation with HOTAIR has its limitations. HOTAIR and MET gene expression showed a remarkable tendency of reverse regulation in adrenocortical carcinoma, cervical squamous cell carcinoma and endocervical adenocarcinoma, lymphoid neoplasm diffuse large b-cell lymphoma, glioblastoma multiforme, head and neck squamous cell carcinoma, kidney renal clear cell carcinoma, acute myeloid leukemia, liver hepatocellular carcinoma, lung adenocarcinoma and uveal melanoma (Fig. 8e). The power of correlation analysis would be more enhanced by expansion of the sample sizes with non-coding gene expression data of public cancer datasets. Besides TCGA, we further analyzed available public research datasets in GEO database. Analysis of available data generated by siRNA knock-down of HOTAIR in an HCC cell line HepG2 [43], (Fig. 8a) and human sarcoma cell lines ES2, A673 and SK-ES (Fig. 8b) supported our in-vitro HOTAIR *knock-down* data in HuH-7 cells [44]. Moreover, data generated by over-expression of HOTAIR in human mesenchymal stem cells was consistent with our data in SNU-449 cells [44], (Fig. 8b, hMSC).

Our study clearly defines the fine tuning of c-Met signaling by HOTAIR to maintain hybrid E/M phenotype which ensures flexibility of commitment and enhances metastatic ability under unfavorable conditions through rate-limiting steps of metastasis. Hybrid phenotype contributes metastatic colonization and may play a crucial role in intra-and extrahepatic metastasis [59]. On the other way around, c-Met activation proceeds E/M spectrum through complete mesenchymal phenotype by suppressing HOTAIR expression. Even this study defines the interplay between two molecules, the detailed molecular interactions are still needed to be investigated. Considering the complex structure and multifunctional behavior, HOTAIR might be regulating c-Met signaling through physically interacting and epigenetic or transcriptional suppression of c-Met and/or its regulators. Further studies are needed to address the molecular cascade between HOTAIR, c-Met and Caveolin-1 in detail.

## Conclusions

In conclusion, HOTAIR over-expression suppresses c-Met expression, activation and also disrupts its organization on plasma membrane by modulating Caveolin-1 expression and activation. HOTAIR over-expression provides an advantage to HCC cells via maintaining hybrid E/M phenotype thereby enhancing survival in adhesion-independent and shear-stressed conditions. HOTAIR overexpression decreases adhesion dependency and proliferation while increasing spheroid formation and improving collective migration abilities and overall improving metastatic potential of HCC cells. Further studies are needed to brighten detailed regulatory signaling cascades and mapping potential targets for therapeutic applications.

## Supplementary information

**Additional file 1: Figure S1.** Includes supplementary data indicated in the text.

**Additional file 2: Supplementary document 1.** Includes primer sequences used in gene expression analysis and catalog numbers of antibodies used in immunoblotting and/or immunofluorescent labeling experiments.

## Data Availability

Eun et al., 2016, performed expression profiling array to identify novel drivers of hepatocellular carcinoma and reveal clinical relevance as early diagnostic and prognostic biomarkers (data accessible at NCBI GEO database (Eun et al., 2016), accession GSE89377). https://www.ncbi.nlm.nih.gov/geo/query/acc.cgi?acc=GSE89377 Wu et al., 2018, performed an integrated proteomic and transcriptomic analysis to examine the overall transcriptomic changes in HepG2 cells after HOTAIR knockdown by RNA sequencing (data accessible at NCBI GEO database (Wu et al., 2018), accession GSE98091). https://www.ncbi.nlm.nih.gov/geo/query/acc.cgi?acc=GSE98091 Shah et al., generated a next-generation RNA-sequencing data of models of Ewing sarcoma with modulated expression (repression with locked nucleic acid antisense oligonucleotides and over-expression with lentiviral transduction) of the lncRNA HOTAIR (data accessible at NCBI GEO database (Shah et al., 2018), accession GSE109483). https://www.ncbi.nlm.nih.gov/geo/query/acc.cgi?acc=GSE109483 The results reported here are in whole or part based upon data generated by the TCGA Research Network: https://www.cancer.gov/tcga.

## Abbreviations

ACC: Adrenocortical carcinoma
BRCA: Breast invasive carcinoma
CESC: Cervical squamous cell carcinoma and endocervical adenocarcinoma
COAD: Colon adenocarcinoma
CTCs: Circulating tumor cells
DLBC: Lymphoid neoplasm diffuse large B-cell lymphoma
EMT: Epithelial to mesenchymal transition
FSS: Fluidic shear stress
GBD: Global Burden of Disease
GBM: Glioblastoma multiforme
HCC: Hepatocellular carcinoma
HNSC: Head and neck squamous cell carcinoma
HOTAIR: HOX Transcript Antisense Intergenic RNA
KICH: Kidney chromophobe
KIRC: Kidney renal clear cell carcinoma
KIRP: Kidney renal papillary cell carcinoma
LAML: Acute myeloid leukemia
LGG: Brain lower grade glioma
LIHC: Liver hepatocellular carcinoma
lncRNA: long non-coding RNA
LUAD: Lung adenocarcinoma
MESO: Mesothelioma
MET: Mesenchymal to epithelial transition
OE: Over-expression
OV: Ovarian serous cystadenocarcinoma
RT-qPCR: Reverse transcriptase-quantitative polymerase chain reaction
RT: Room temperature
RTCA: Real time cell analysis system
TCGA: The Cancer Genome Atlas
UVM: Uveal melanoma
