# Supplementary figures and images for: lncRNA HOTAIR overexpression induced downregulation of c-Met signaling promotes hybrid epithelial/mesenchymal phenotype in hepatocellular carcinoma cells
Source: Cell Commun Signal. 2020 Jul 11;18:110. doi: 10.1186/s12964-020-00602-0 (PMC7353702; doi:10.1186/s12964-020-00602-0)

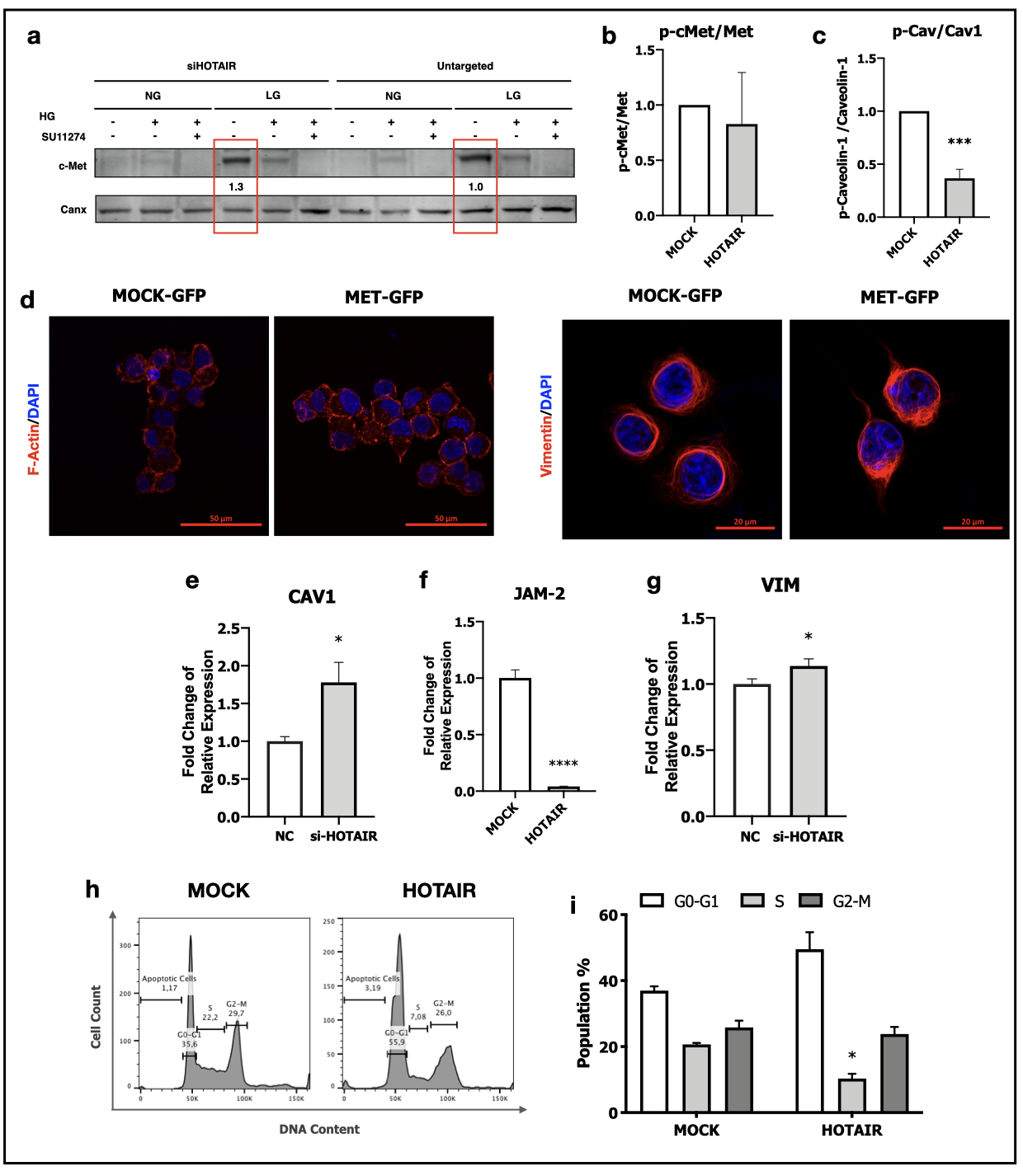

Supplement: Supplementary file 2 — Additional file 1: Figure S1. Includes supplementary data indicated in the text. [file 12964_2020_602_MOESM1_ESM.tiff]
