# Supplementary material for: lncRNA HOTAIR overexpression induced downregulation of c-Met signaling promotes hybrid epithelial/mesenchymal phenotype in hepatocellular carcinoma cells
Source: Cell Commun Signal. 2020 Jul 11;18:110. doi: 10.1186/s12964-020-00602-0 (PMC7353702; doi:10.1186/s12964-020-00602-0)
Supplement: Supplementary file 3 — Additional file 2: Supplementary document 1. Includes primer sequences used in gene expression analysis and catalog numbers of antibodies used in immunoblotting and/or immunofluorescent labeling experiments. [file 12964_2020_602_MOESM2_ESM.docx]

**Supplementary Document 1.**

List of primers used in RT-qPCR analysis:

| HOTAIR | Forward: | GGTAGAAAAAGCAACCACGAAGC |
| --- | --- | --- |
|  | Reverse: | ACATAAACCTCTGTCTGTGAGTGCC |
| MET | Forward: | GTCAGCCTTGTCCCTCCTTC |
|  | Reverse: | GTCCTGCAGTCAATGCCTCT |
| CAV-1 | Forward: | TGAGCGAGAAGCAAGTGTACG |
|  | Reverse: | CTGTGTGTCCCTTCTGGTTCT |
| VIM | Forward: | CCTTGACATTGAGATTGCCACCTA |
|  | Reverse: | TCATCGTGATGCTGAGAAGTTTCG |
| CDH-1 | Forward: | TACACTGCCCAGGAGCCAGA |
|  | Reverse: | TGGCACCAGTGTCCGGATTA |
| CDH-3 | Forward: | ATTGGACCATCACTCGGCTTA |
|  | Reverse: | CACACTGGCAAACCTTCACG |
| JAM2 | Forward: | TGGAAGAAACTGGGTCGGAG |
|  | Reverse: | TTCCCCGCATCACTTCTTGT |
| RPL41 | Forward: | GAAACCTCTGCGCCATGA |
|  | Reverse: | TCTTTCTTCTTTTGCGCTTCA |
| GAPDH | Forward: | GAAGGTGAAGGCGGAGTC |
|  | Reverse: | GAAGATGGTGATGGGATTTC |
| ITGA6 | Forward: | AAGCTCTCGTAGGCGAGTGC |
|  | Reverse: | TCTGGAAACGTTGCAATCAG |
| ITGB1 | Forward: | TGTGAATGCAGCACAGATGA |
|  | Reverse: | AGACACCACACTCGCAGATG |
| ITGA4 | Forward: | TCGCCAACGCTTCAGTGATCAATCC |
|  | Reverse: | TCTATGCCCACAAGTCACGATGGA |
| ITGB4 | Forward: | AGTGAAGAGGTGCGGAGT |
|  | Reverse: | GTTCTGCCCCATCTTCTTGA |

List of antibodies used in immunoblotting and immunofluorescent label:

| Cell Signaling | 9272 | Akt |
| --- | --- | --- |
| Cell Signaling | 4060 | Phospho-Akt (Ser-473) |
| Santa Cruz Biotechnologies | Sc-7963 | beta-Catenin |
| Santa Cruz Biotechnologies | Sc-11397 | Calnexin |
| Santa Cruz Biotechnologies | Sc-894 | Caveolin-1 |
| BD Biosciences | BD610058 | Caveolin-1 |
| BD Biosciences | BD 611339 | Phospho-caveolin-1 (Tyr-14) |
| Santa Cruz Biotechnologies | Sc-11397 | Calnexin |
| Cell Signaling | 13116 | N-Cadherin |
| Cell Signaling | 3195 | E-cadherin |
| Santa Cruz Biotechnologies | Sc-8426 | E-cadherin |
| Cell Signaling | 9101 | Phospho-Erk 1/2 p44/42 (MAPK) (Thr202/Tyr204) |
| Cell Signaling | 4377 | Phospho-Erk 1/2 p44/42 (MAPK) (Thr202/Tyr204) |
| Cell Signaling | 4696 | Erk 1/2 p44/42 |
| Cell Signaling | 3127 | Met |
| Santa Cruz Biotechnologies | Sc-161 | Met |
| Cell Signaling | 3129 | Phospho-Met (Y-1234/1235) |
| Cell Signaling | 4113 | pSTAT3 (Y705) |
| Cell Signaling | 482 | STAT3 |
| Cell Signaling | 4904 | STAT3 |
| Cell Signaling | 5741 | Vimentin |
| Santa Cruz Biotechnologies | Sc-373717 | Vimentin |
| Santa Cruz Biotechnologies | Sc-1615 | Beta-Actin |
| Invitrogen | A-11005 | Alexa594-conjugated goat anti-mouse secondary antibody |
| Invitrogen | A-11037 | Alexa594-conjugated goat anti-rabbit secondary antibody |

List of lentiviral and retroviral vectors used in generation of stable cell clones:

1. LZRS-HOTAIR was a gift from Howard Chang (Addgene plasmid #26110; <http://n2t.net/addgene:26110>; RRID: Addgene_26110).
2. LZRS-IresGFP was a gift from Lynda Chin (Addgene plasmid #21961; http://n2t.net/addgene:21961; RRID: Addgene_21961).
3. pMD2.G was a gift from Didier Trono (Addgene plasmid #12259; http://n2t.net/addgene:12259; RRID: Addgene_12259).
4. pUMVC was a gift from Bob Weinberg (Addgene plasmid # 8449).
5. pLenti-MetGFP was a gift from David Rimm (Addgene plasmid #37560; http://n2t.net/addgene:37560; RRID: Addgene_37560).
6. pLenti-CMV-GFP Puro (658-5) was a gift from Eric Campeau & Paul Kaufman (Addgene plasmid #17448; http://n2t.net/addgene:17448; RRID: Addgene_17448).
7. pRSV-Rev (Addgene plasmid #12253; http://n2t.net/addgene:12253; RRID: Addgene_12253).
8. pMDLg/pRRE was a gift from Didier Trono (Addgene plasmid #12251; http://n2t.net/addgene:12251; RRID: Addgene_12251).
